# Integrating Conformational Dynamics and Perturbation-Based Network Modeling for Mutational Profiling of Binding and Allostery in the SARS-CoV-2 Spike Variant Complexes with Antibodies: Balancing Local and Global Determinants of Mutational Escape Mechanisms

**DOI:** 10.3390/biom12070964

**Published:** 2022-07-10

**Authors:** Gennady Verkhivker, Steve Agajanian, Ryan Kassab, Keerthi Krishnan

**Affiliations:** 1 Keck Center for Science and Engineering, Graduate Program in Computational and Data Sciences, Schmid College of Science and Technology, Chapman University, Orange, CA 92866, USA; agaja102@mail.chapman.edu (S.A.); rkassab@chapman.edu (R.K.); kekrishnan@chapman.edu (K.K.); 2 Department of Biomedical and Pharmaceutical Sciences, Chapman University School of Pharmacy, Irvine, CA 92618, USA

**Keywords:** SARS-CoV-2 spike protein, ultrapotent antibodies, antibody escape, protein stability, mutational profiling, conformational dynamics, spike plasticity, dynamic network modeling, binding hotspots, allosteric communication

## Abstract

In this study, we combined all-atom MD simulations, the ensemble-based mutational scanning of protein stability and binding, and perturbation-based network profiling of allosteric interactions in the SARS-CoV-2 spike complexes with a panel of cross-reactive and ultra-potent single antibodies (B1-182.1 and A23-58.1) as well as antibody combinations (A19-61.1/B1-182.1 and A19-46.1/B1-182.1). Using this approach, we quantify the local and global effects of mutations in the complexes, identify protein stability centers, characterize binding energy hotspots, and predict the allosteric control points of long-range interactions and communications. Conformational dynamics and distance fluctuation analysis revealed the antibody-specific signatures of protein stability and flexibility of the spike complexes that can affect the pattern of mutational escape. A network-based perturbation approach for mutational profiling of allosteric residue potentials revealed how antibody binding can modulate allosteric interactions and identified allosteric control points that can form vulnerable sites for mutational escape. The results show that the protein stability and binding energetics of the SARS-CoV-2 spike complexes with the panel of ultrapotent antibodies are tolerant to the effect of Omicron mutations, which may be related to their neutralization efficiency. By employing an integrated analysis of conformational dynamics, binding energetics, and allosteric interactions, we found that the antibodies that neutralize the Omicron spike variant mediate the dominant binding energy hotpots in the conserved stability centers and allosteric control points in which mutations may be restricted by the requirements of the protein folding stability and binding to the host receptor. This study suggested a mechanism in which the patterns of escape mutants for the ultrapotent antibodies may not be solely determined by the binding interaction changes but are associated with the balance and tradeoffs of multiple local and global factors, including protein stability, binding affinity, and long-range interactions.

## 1. Introduction

The rapidly growing body of structural, biochemical, and functional studies established that the mechanism of SARS-CoV-2 infection may involve conformational transitions between distinct functional forms of the SARS-CoV-2 viral spike (S) glycoprotein [1,2,3,4,5,6,7,8,9]. The S protein consists of a conformationally adaptive amino (N)-terminal S1 subunit and structurally rigid carboxyl (C)-terminal S2 subunit, where S1 includes an N-terminal domain (NTD), the receptor-binding domain (RBD), and two structurally conserved subdomains, SD1 and SD2, which coordinate the protein response to binding partners and regulate the interactions with the host cell receptor ACE2. Conformational plasticity of the SARS-CoV-2 S protein is exemplified by spontaneous transitions from the closed state to the open state accompanied by large-scale movements of the RBDs that can spontaneously fluctuate between the “RBD-down” and “RBD-up” positions, where binding to the host cell receptor ACE2 preferentially stabilizes the receptor-accessible “up” conformation [1,2,3,4,5,6,7,8,9,10,11,12]. The cryo-EM experimental tools have been deployed at an unprecedented speed to characterize the dynamic structural changes in the SARS-CoV-2 S protein, revealing a spectrum and atomic details of the prefusion S conformations that included various forms of the closed “RBD-down” state, “partially open” trimers with only one or two RBDs in the “up” conformation, and “open” trimers with all three RBDs in the “up” position [5,6,7,8,9,10,11,12,13,14]. The cryo-EM and tomography tools examined conformational flexibility and distribution of the S trimers in situ on the virion surface showing that the underlying physical mechanism of spontaneous conformational changes between different functional open and closed S states and the intrinsic properties of the conformational landscapes for SARS-CoV-2 S trimers are preserved in different biological environments [15]. Single-molecule Fluorescence (Förster) Resonance Energy Transfer (smFRET) studies have captured the intrinsically dynamic nature of the SARS-CoV-2 S trimer, suggesting that conformational selection and receptor-induced structural adaptation of the S states can work synchronously, leading to diverse mechanisms for antibody-induced neutralization [16]. Biophysical studies demonstrated that conformational dynamics and allosteric regulation of the S protein are intrinsically related, in which allosteric modulation of the RBD equilibrium can be a critical component of the SARS-CoV-2 virus adaption strategies [17]. Allosteric modulation of the conformational dynamics and ligand-induced population shifts in the S protein was quantified using an smFRET imaging assay showing that antibodies may allosterically promote a shift in the RBD equilibrium toward the up conformation, enhancing ACE2 binding [18]. The SARS-CoV-2 variants of concern (VOCs) are characterized by the enhanced transmissibility and infectivity profiles, promoting immune evasion and partial vaccine escape [19,20,21,22,23,24,25,26,27,28]. The Omicron variants (B.1.1.529, BA.1, BA.1.1, BA.2, BA.3, BA.4, and BA.5 lineages) [29,30,31,32,33,34] can facilitate the evasion of immune responses induced by vaccination and confer resistance to a wide spectrum of neutralizing antibodies that are one of the significant components for adaptive immunity against viruses. Structural studies of the Omicron variant suggested that evolutionary pressure invokes a complex interplay of thermodynamic factors between mutations that increase affinity for the ACE2 with other RBD modifications that disfavor ACE2 binding but facilitate immune escape [29,30]. These investigations suggested that immune evasion may be a primary driver of Omicron evolution that sacrifices some ACE2 affinity enhancement substitutions to optimize immune-escaping mutations. Using surface plasmon resonance (SPR) to measure binding of the RBD to human and mouse ACE2 receptors, it was demonstrated that the Omicron RBD has a 2.4-fold increased binding affinity to human ACE2 [31]. A detailed thermodynamic and kinetics analysis of the effect of five common S-RBD mutations (K417N, K417T, N501Y, E484K, and S477N) on the binding affinity with ACE2 showed that the Omicron mutations N501Y and S477N enhance transmission by enhancing binding, while K417N facilitates immune escape [32]. The antibody-escaping mutation profiles revealed that single Omicron mutations can impair neutralizing antibodies of different classes, particularly for antibodies targeting the ACE2-binding epitopes that are escaped by the single mutations K417N, G446S, E484A, and Q493R [33]. By evaluating monoclonal antibodies against all known epitope clusters on the S protein, the activity of 17 out of the 19 currently authorized or approved antibodies were impaired against the Omicron variant, revealing that mutations S371L, N440K, G446S, and Q493R can confer greater antibody resistance [34]. The structure–function investigations of the SARS-CoV-2 S Omicron variant in various functional states and complexes with antibodies reinforced and further detailed the diversity of neutralization escape mechanisms that can be determined by multiple fitness trade-offs balancing the tendency to evolve mutations evading antibodies with mutational changes that preserve or enhance binding affinity with ACE2 [35,36,37,38,39,40]. Structural analysis of the SARS-CoV-2 Omicron S protein states showed that, unlike other VOCs implicated in the enhanced viral transmissibility through mutation-induced stabilization of the open S states, the Omicron variant can lead to the increased thermodynamic stabilization of the closed state and promote immune evasion by occluding immunogenic sites [40]. These studies demonstrated that the intrinsic conformational flexibility of the S protein, which is distinctly controlled by mutations, is an important contributing factor of antibody escape induced by the Omicron variant.

Although the enhanced binding interactions of the S-RBD Omicron protein with ACE2 are often attributed to the greater infectivity of the Omicron variant, recent biophysical studies emphasized the role of mechanical stability as an additional mechanism for mediating stronger virus–cell interactions and immune evasion [41,42,43]. Atomic force microscopy and computer simulations examined the stability effects of several variants on the kinetic and thermodynamic properties of the RBD–ACE2 complex formation, showing that the RBD mutations in the different variants led in all cases to both the higher stability and affinity of the RBD–ACE2 complex [41]. Single-molecule experiments that quantifies the molecular stiffness of the SARS-CoV-2 S proteins demonstrated that the S protein can exploit mechanical force to enhance its recognition of ACE2 and subsequently accelerate S1/S2 detachment for effective invasion into host cells [42]. Another fascinating biophysical study employed single-molecule force spectroscopy techniques to quantify the force stability of the RBD–ACE2 interactions under physiological conditions, revealing the higher mechanical stability, greater binding free energy, and a lower dissociation rate for the SARS-CoV-2 S protein as compared to SARS-CoV-1 [43]. These studies established a synergistic role of mechanical forces, protein stability effects, and binding interaction factors in mediating the spike association with the host receptor and antibodies that collectively control the virus fitness advantage and immune escape mechanisms.

To understand the antigenic anatomy of the SARS-CoV-2 S protein, and the molecular mechanisms of SARS-CoV-2 neutralization, several studies examined the diversity of the binding epitopes in the structures of the antibody complexes and presented a detailed classification of these antibodies into distinct categories [44,45,46,47,48]. Classes of neutralizing antibodies that are characterized by direct ACE2 competition via binding RBD-up/down conformations exhibit the highest potency but often suffer from the greatest sensitivity to viral escape, whereas antibodies targeting cryptic epitopes that do not interfere with ACE2 binding are characterized by a reduced potency but displaying a greater breadth and tolerance to RBD mutations. Combinations and synergistic cocktails of different antibody classes simultaneously targeting the conserved and more variable SARS-CoV-2 RBD epitopes can provide more efficient cross-neutralization effects and yield resilience against mutational escape [49,50]. Using an arsenal of biophysical tools, including antigen-based flow-sorting and live virus neutralization assays, a recent study identified four antibodies (A19-46.1, A19-61.1, A23-58.1, and B1-182.1) that target the RBD and neutralize the SARS-CoV-2 WA-1 original virus strain, while also maintaining potent neutralizing activity against 23 variants, including B.1.1.7, B.1.351, P.1, B.1.429, B.1.526, B.1.529.1, B.1.617.1, and B.1.617.2 VOCs. [51]. The cryo-EM reconstructions for structures of the A23-58.1 and B1-182.1 bound to the original Wuhan or WA1/2020 strain (S-WA1) revealed that the antibodies bind to the S protein with all RBDs in the up position. An impressive structure–function tour-de-force investigation of the impact of the SARS-CoV-2 S mutations on the binding and neutralization of monoclonal antibodies confirmed that many potent antibodies targeting the spike RBD experienced a significant loss of binding for the B.1.1.529 variant [52]. Using functional assays and cryo-EM structures of the S Omicron complexes with a large panel of antibodies, this study identified that only A23-58.1, B1-182.1, COV2-2196, S2E12, A19-46.1, S309, and LY-CoV1404 maintain substantial neutralization against the Omicron variant. Class I B1-182.1 and class II A19-46.1 antibodies revealed potent neutralization of B.1.1.529, while class III and IV antibodies that bind outside of the ACE2-binding surface (A19-61.1, COV2-2130, S309, and LY-CoV1404) can tolerate individual B.1.1.529 substitutions [52]. The structure of the ternary complex of the Omicron complex with a combination of B1-182.1 and A19-46.1 antibodies targeting different binding epitopes and trapping three RBD-up S conformations suggested a mechanism of the observed synergistic increase in neutralization potency compared with that of the individual antibodies [52]. Analysis of the neutralization profiles for a broad panel of antibodies against the Omicron sub-lineages showed that while most antibodies lost neutralizing activity, some display a unique Omicron escape potential, reflecting antigenic differences [53]. The effect of individual and combined mutations that convergently appeared in different lineages was examined showing that the RBD sites more dispensable for binding to ACE2 (particularly E484 and S494) can be hotspots for immune evasion, and in combination with mutations that promote ACE2 binding, such as N501Y, can increase escape neutralizing-antibody responses [54]. The recent investigation identified two highly conserved cryptic regions on the S-RBD Omicron that are simultaneously and synergistically recognized by a bispecific single-domain antibody [55]. The recent pioneering discoveries of broadly neutralizing antibodies that are effective against multiple VOCs showed that they target highly conserved binding epitopes or conserved cryptic regions, where the antibody-interacting RBD residues are rarely mutated in the GISAID database [56]. Collectively, structural, functional, and biophysical studies revealed the diversity of mechanistic scenarios underlying antibody binding and catalogued the RBD escape mutations for a wide range of antibodies, revealing distinct signatures of antibody-resistant mutational hotspots.

Computer simulations provided important atomistic and mechanistic insights into understanding the dynamics and function of the SARS-CoV-2 glycoproteins. Molecular dynamics (MD) simulations of the SARS-CoV-2 S proteins and mutants detailed conformational changes and diversity of ensembles, demonstrating enhanced functional and structural plasticity of the S proteins [57,58,59,60,61,62,63,64,65]. All-atom MD simulations of the full-length SARS-CoV-2 S glycoprotein embedded in the viral membrane, with a complete glycosylation profile, were performed by Amaro and coworkers, providing an unprecedented level of detail about the conformational landscapes of the S proteins in the physiological environment [57]. Another landmark study from this laboratory reported 130 µs of weighted ensemble simulations of the fully glycosylated S ectodomain and statistical characterization of more than 300 kinetically unbiased RBD-opening pathways [58]. Together with the cryo-electron microscopy data and biolayer interferometry experiments, these integrative studies revealed a gating role for the *N*-glycan at position N343 in facilitating RBD opening and suggested that allosteric mechanisms are in play to control the balance between the closed and open S conformations [58]. MD simulations of the S-protein in solution and targeted simulations of conformational changes between the open and closed forms revealed the key electrostatic interdomain interactions mediating the protein stability and kinetics of the functional spike states [64]. MD simulations characterized the conformational landscapes of the full-length S protein trimers detailing conformational transitions between functional states and unveiling previously unknown cryptic pockets [65]. Our studies revealed that the SARS-CoV-2 S protein can function as an allosteric regulatory machinery that can exploit the intrinsic plasticity of functional regions controlled by stable allosteric hotspots to modulate specific regulatory and binding functions [66,67,68,69,70,71,72]. A number of computational studies employed atomistic simulations and binding energy analysis to examine the interactions between the S-RBD Omicron and the ACE2 receptor. Markov state modeling of the conformational states and binding free energy calculations helped identified the key mutational sites (S477N, G496S, Q498R, and N501Y) responsible for the enhanced binding of ACE2 by the Omicron RBD [73]. The dynamics and energetics of the interactions between the Omicron RBD and ACE2 was examined using MD simulations, confirming the greater affinity of the Omicron variant as compared to the original S-WA1 strain [74]. Computational mutagenesis and binding free energy analysis showed that the RBD Omicron binds ACE2 approximately 2–3 times stronger than the original S-WA1 protein, where the three mutational sites T478K, Q493K, and Q498R enhance the binding affinity through more favorable electrostatic interactions [75]. All-atom MD simulations of the S Omicron trimer and the Omicron RBD–ACE2 complexes suggested that the Omicron mutations may have evolved to inflict a greater infectivity using a combination of more efficient RBD opening, the increased binding affinity with ACE2, and optimized capacity for antibody escape [76]. The role of the electrostatic potentials in the RBD–ACE2 binding suggested that the RBD mutations increasing the electropositive-charged residues may contribute to the enhanced transmission by favoring binding with the electronegative-charged ACE2 receptors [77]. The effect of nonadditive, epistatic relationships among RBD mutations was assessed using protein structure modeling by comparing the effects of all single mutants at the RBD–ACE2 interfaces for the Omicron variants, showing that structural constraints on the RBD can curtail the virus evolution for a more complete vaccine and antibody escape [78]. By employing the conformational ensembles of the S-RBD Omicron variant complexes with ACE2, we recently performed simulations and mutational scanning of the interfacial RBD residues, showing that N501Y is the critical binding affinity hotspot in the S Omicron RBD complex with ACE2, while hotspots Q493R, G496S, and Q498R anchor the key interfacial clusters responsible for binding with ACE2 [79].

In the current study, we expanded on our previous investigations and employed a battery of several computational approaches to examine the binding mechanisms of the S-RBD Omicron protein with a set of ultra-potent antibodies that elicit significant neutralization of the Omicron variant and are resistant to or limit the antibody escape. We combined all-atom MD simulations, mutational scanning of protein folding stability and binding, and perturbation-based network profiling of allosteric interactions in the S-RBD and S-RBD Omicron structures with a panel of cross-reactive single antibodies (B1-182.1 and A23-58.1) as well as combinations (A19-61.1/B1-182.1 and A19-46.1/B1-182.1). Using these approaches, we quantify both the local and global effects of the RBD mutations, identify protein stability centers, characterize binding energy hotspots, and predict the allosteric control points of long-range interactions in the S-RBD Omicron complexes with the antibodies. A network-based perturbation approach for mutational profiling of allosteric residues potentials is proposed that evaluates the effect of antibody binding on modulation of allosteric interactions in the studied S-RBD complexes. Through analysis of the conformational landscapes and systematic mutational profiling of binding, protein stability, and allostery in the RBD–antibody complexes, this study quantifies the structural and energetic factors of the binding mechanism that can contribute to the experimentally observed pattern of limited antibody escape. Consistent with the experimental evidence, mutational profiling reveals that local binding interactions and long-range allosteric communications in the complexes are generally tolerant to the Omicron mutations. The results show that the dominant binding energy hotspots and allosteric centers of long-range interactions in the S-RBD Omicron complexes correspond to the same group of conserved RBD residues—Y449, Y453, L455, F486, Y489, and F490—that are also vital for the RBD stability and ACE2 binding. This study suggests that the antibody escape patterns may not be solely determined by the binding interaction changes but are affected by both local and global factors. The findings of this investigation suggest a plausible mechanism in which binding of the antibodies to the conserved and “mutation-protected” RBD regions may narrow the “ evolutionary path” for the virus, thereby allowing for the neutralization efficiency against the Omicron variant. We suggest that the antibody escape mechanisms are associated with the multiple fitness tradeoffs balancing the conformational plasticity and local binding interactions, the RBD protein stability, and the functional control of global structural changes mediated by allosteric hotspots.

## 2. Materials and Methods

### 2.1. Molecular Dynamics Simulations

All structures were obtained from the Protein Data Bank [80]. During structure preparation stage, protein residues in the crystal structures were inspected for missing residues and protons. Hydrogen atoms and missing residues were initially added and assigned according to the WHATIF program web interface [81]. The missing loops in the studied cryo-EM structures of the SARS-CoV-2 S protein were reconstructed and optimized using the template-based loop prediction approaches ModLoop [82] and ArchPRED server [83]. The side chain rotamers were refined and optimized by SCWRL4 tool [84]. The protein structures were then optimized using atomic-level energy minimization with composite physics and knowledge-based force fields as implemented in the 3Drefine method [85]. The atomistic structures from the simulation trajectories were further elaborated by adding N-acetyl glycosamine (NAG) glycan residues and were optimized. We performed 10 independent all-atom MD simulations (500 ns each simulation) for each of the following S-RBD complexes: S-RBD with A23-58.1 (pdb id 7LRS), S-RBD with B1-182.1 (pd id 7MLZ), S-RBD with A19-61.1/B1-182.1 (pdb id 7TBF), and S-RBD Omicron with A19-46.1/B1-182.1 (pdb id 7U0D) (Table 1), using the NAMD 2.13-multicore-CUDA package [86] with a CHARMM36 force field [87]. The all-atom additive CHARMM36 protein force field can be obtained from http://mackerell.umaryland.edu/charmm_ff.shtml. (accessed on 17 March 2022). For each system, multiple independent simulations were initiated from the same atomic coordinates obtained from the experimental structures but with randomized initial velocities. The equilibrium ensembles for the analysis were derived by aggregating the 10 independent MD trajectories for every system.

The structures of the SARS-CoV-2 S-RBD complexes were prepared in Visual Molecular Dynamics (VMD 1.9.3) [88] by placing them in a TIP3P water box with 20 Å thickness from the protein. Assuming normal charge states of the ionizable groups, corresponding to a pH = 7, sodium (Na^+^) and chloride (Cl^−^) counter-ions were added to achieve charge neutrality and a salt concentration of 0.15 M NaCl was maintained. All Na^+^ and Cl^−^ ions were placed at least 8 Å away from any protein atoms and from each other. The long-range non-bonded van der Waals interactions were computed using an atom-based cutoff of 12 Å with the switching function beginning at 10 Å and reaching zero at 14 Å. The SHAKE method was used to constrain all bonds associated with hydrogen atoms. Simulations were run using a leap-frog integrator with a 2-fs integration time step. The ShakeH algorithm of NAMD was applied for water molecule constraints. The long-range electrostatic interactions were calculated using the particle mesh Ewald method [89] with a real space cut-off of 1.0 nm and a fourth order (cubic) interpolation. Simulations were performed under NPT ensemble with a Langevin thermostat and Nosé–Hoover Langevin piston at 310 K and 1 atm. The damping coefficient (gamma) of the Langevin thermostat was 1/ps. The Langevin piston Nosé–Hoover method in NAMD is a combination of the Nose–Hoover constant pressure method [90] with piston fluctuation control implemented using Langevin dynamics [91,92]. Energy minimization after addition of solvent and ions was conducted using the steepest descent method for 100,000 steps. All atoms of the complex were first restrained at their crystal structure positions with a force constant of 10 Kcal mol^−1^ Å^−2^. Equilibration was done in steps by gradually increasing the system temperature in steps of 20 K, starting from 10 K until 310 K, and at each step 1 ns equilibration was done keeping a restraint of 10 Kcal mol^−1^ Å^−2^ on the protein C_α_ atoms. After the restrains on the protein atoms were removed, the system was equilibrated for additional 10 ns. An NPT production simulation was run on the equilibrated structures for 500 ns keeping the temperature at 310 K and at a constant pressure (1 atm).

### 2.2. Distance Fluctuations Stability and Communication Analysis

We employed distance fluctuation analysis of the simulation trajectories to compute the residue-based stability profiles. The fluctuations of the mean distance between each pseudo-atom belonging to a given amino acid and the pseudo-atoms belonging to the remaining protein residues were computed. The fluctuations in the mean distance between a given residue and all other residues in the ensemble were converted into distance fluctuation stability indexes that measure the energy cost of the residue deformation during simulations [93,94,95,96]. The distance fluctuation stability index for each residue is calculated by averaging the distances between the residues over the simulation trajectory using the following expression:

(1) $$k_{i}=\frac{3k_{B}T}{{\langle (d_{i}-\langle d_{i}\rangle )}^{2}\rangle }$$

(2) $$d_{i}=\langle d_{ij}\rangle_{j*}$$

where $d_{ij}$ is the instantaneous distance between residue i and residue j; $k_{B}$ is the Boltzmann constant; T = 300 K; 〈〉 denotes an average taken over the MD simulation trajectory; and $d_{i}=\langle d_{ij}\rangle_{j*}$ is the average distance from residue i to all other atoms j in the protein (the sum over $j_{*}$ implies the exclusion of the atoms that belong to the residue i). The interactions between the $C_{\alpha }$ atom of residue i and the $C_{\alpha }$ atom of the neighboring residues i − 1 and i + 1 are excluded in the calculation since the corresponding distances are constant. The inverse of these fluctuations yields an effective force constant *k_i_* that describes the ease of moving an atom with respect to the protein structure. The dynamically correlated residues whose effective distances fluctuate with low or moderate intensity are expected to communicate over long distances with a higher efficiency than the residues that experience large fluctuations. Our previous studies showed that residues with a high value of these indexes often serve as protein stability centers and regulatory points of allosteric interactions and communications, whereas small values of the distance fluctuation stability index are typically indicative of highly dynamic fluctuating sites [67,68,69]. The python scripts and tools used for calculation of the distance fluctuations are reported in Supplementary Materials Information and require the mdtraj, matplotlib, and cython modules.

### 2.3. Mutational Scanning and Sensitivity Analysis

Mutational sensitivity scanning of the SARS-CoV-2 S-RBD and S-RBD Omicron complexes with the studied antibodies were done using the BeAtMuSiC approach [97,98,99] and the webserver at http://babylone.ulb.ac.be/beatmusic/index.php (accessed on 25 April 2022). The BeAtMuSiC server allows for a systematic scan of all mutations in a protein chain (or group of chains), or at the protein–protein interface. Each binding epitope residue for the studied complexes was systematically mutated using all possible substitutions and the corresponding free energy changes were computed. The BeAtMuSiC approach is based on statistical potentials describing the pairwise inter-residue distances, backbone torsion angles, and solvent accessibilities, and considers the mutational effects on the binding interactions and the overall thermal stability of the complex. The binding free energy of the protein–protein complex can be expressed as the difference in the folding free energy of the complex and folding free energies of the two protein binding partners:

(3) $$\Delta G_{bind}=G^{com}-G^{A}-G^{B}$$

The change in binding energy due to a mutation was calculated then as the following:

(4) $$\Delta \Delta G_{bind}=\Delta G_{bind}^{mut}-\Delta G_{bind}^{wt}{{}_{}}^{}$$

We leveraged rapid calculations using the BeAtMuSiC server to compute the ensemble-averaged binding free energy changes using equilibrium samples from MD trajectories. The reported binding free energy changes are obtained by averaging the BeAtMuSiC results over 100 equilibrium conformations for each of the studied systems.

### 2.4. Network Analysis and Perturbation-Based Mutational Profiling of Allosteric Propensities

A graph-based representation of the protein structures [100,101] is used to represent residues as network nodes and the inter-residue edges to describe non-covalent residue interactions. The network edges that define residue connectivity are based on non-covalent interactions between the residue side-chains. We constructed the residue interaction networks using dynamic correlations [102] that yield robust network signatures of long-range couplings and communications. The Residue Interaction Network Generator (RING) program was employed for the initial generation of residue interaction networks based on the single structure [103] and the conformational ensemble [104], where edges have an associated weight reflecting the frequency in which the interaction present in the conformational ensemble. The residue interaction network files in xml format were obtained for all structures using the RING v3.0 webserver freely available at https://ring.biocomputingup.it/submit (accessed on 15 May 2022). Network graph calculations were performed using the python package NetworkX [105]. Using the constructed protein structure networks, we computed the residue-based betweenness parameter. The short path betweenness centrality of residue i is defined to be the sum of the fraction of the shortest paths between all pairs of residues that pass through residue i:

(5) $$C_{b}(n_{i})=\sum_{j<k}^{N}\frac{g_{jk}(i)}{g_{jk}}$$

where $g_{jk}$ denotes the number of shortest geodesics paths connecting j and *k*; and $g_{jk}(i)$ is the number of shortest paths between residues j and *k* passing through the node $n_{i}$. The betweenness centrality metric is also computed by evaluating the average shortest path length (ASPL) change by systematically removing individual nodes [106,107]. The following Z-score is then calculated:

(6) $$Z_{i}=\frac{B_{k}-\langle B\rangle }{\sigma }$$

Through mutation-based perturbations of the protein residues we computed the dynamic couplings of residues and changes in the average short path length (ASPL) averaged over all modifications in a given position. The change in ASPL upon mutational changes of each node is inspired and reminiscent of the calculation proposed to evaluate residue centralities by systematically removing nodes from the network.

(7) $$\Delta L_{i}=\langle {\left|\left|\Delta L_{i}^{node}\left(j\right)\right|\right|}^{2}\rangle$$

where *i* is a given site; *j* is a mutation; and 〈⋯〉 denotes averaging over mutations. $\Delta L_{i}^{node}\left(j\right)$ describes the change in ASPL upon mutation j in a residue node i. $\Delta L_{i}$ is the average change in ASPL induced by all mutations of a given residue. The Z-score is then calculated for each node as follows:

(8) $$Z_{i}=\frac{\Delta L_{i}-\langle \Delta L\rangle }{\sigma }$$

where 〈ΔL〉 is the change in ASPL under mutational scanning averaged over all protein residues in the S-RBD and σ is the standard deviation. The ensemble-averaged Z–score ASPL changes were computed from a network analysis of the conformational ensembles using 1000 snapshots of the simulation trajectory for the native protein system.

## 3. Results and Discussion

### 3.1. Structural Analysis of the S-RBD Complexes with Antibodies

**Figure 1:**
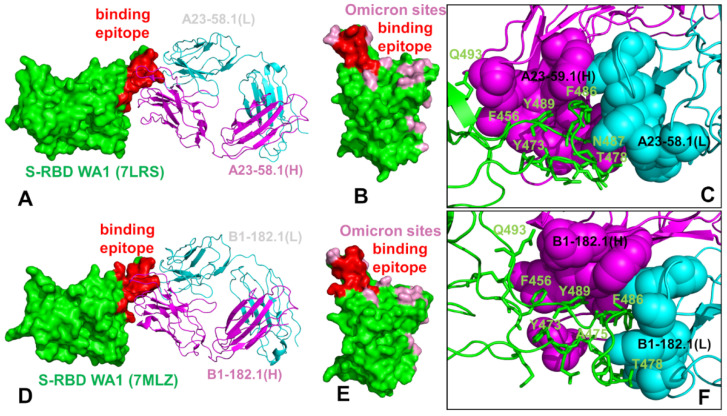
Structural organization of the SARS-CoV-2 RBD complexes with the A23-58.1 and B1-182.1 antibodies. (**A**) The cryo-EM structure of the S-RBD WA1 complex with A23-58.1. The S-RBD is in green, the binding epitope residues are colored in red, and the antibody is shown in ribbons. The heavy chain of A23-58.1 is in magenta and the light chain in cyan. (**B**) The S-RBD is shown in green with the binding epitope residues in red and the RBD sites of the Omicron mutations (G339, S371, S373, S375, K417, N440, G446, S477, T478, E484, Q493, G496, Q498, N501, and Y505) are colored in pink. (**C**) A detailed closeup of the interacting residues in the binding interface of the S-RBD complex with A23-58.1. The S-RBD binding residues are shown in green sticks and annotated. The contact sites of the heavy antibody chain are in magenta spheres and in cyan spheres for the light chain residues. (**D**) The cryo-EM structure of the S-RBD WA1 complex with B1-182.1 antibody. The S-RBD is in green, the binding epitope residues are colored in red, and the antibody is shown in ribbons. The heavy chain of is in magenta and the light chain in cyan. (**E**) The S-RBD green surface with the binding epitope residues in red and the RBD sites of Omicron mutations in pink. (**F**) A closeup of the interacting residues in the binding interface of the S-RBD complex with B1-182.1. The S-RBD-binding residues are shown in green sticks and annotated, and the contact sites on the antibody are shown in magenta and cyan spheres for the heavy and light chains, respectively.

We began with the structural analysis of the S-RBD binding with single antibodies A23-58.1 (Figure 1A–C) and B1-182.1 (Figure 1D–F). The cryo-EM structures of the S-RBD WA1 complexes showed that both antibodies adapt the mode of binding by directly blocking the interaction of the RBD with ACE2 and could be classified as either class I (ACE2 blocking, binding RBD up only) or class II RBD antibodies (ACE2 blocking, binding RBD up or down). A23-58.1 an B1-182.1 bind to an invariant region of the RBD tip, with the binding epitope formed by residues K417, L455, F456, K458, Y473, Q474, A475,G476, N477, K478, C480, E484, G485, F486, N487, Y489, and Q493 (Figure 1A,D). Mapping of the binding epitope residues together with the sites of Omicron mutations (Figure 1B,E) highlighted a minor overlap, where only the K417, N477, and K478 positions engage in the interactions with the antibodies. The interaction details of the antibody–RBD interface showed that the RBD tip region binds to a “crater-like” region formed by the complementarity-determining regions (CDRs) of the antibodies (Figure 1C,F). The key intermolecular interactions are formed between the aromatic RBD residues F456, F486, and Y489 and a group of hydrophobic sites in the heavy and light chains of the antibodies. The most prominent contacts are mediated by F486 deeply protruding into the antibody “crater” and surrounded by P95, F100 (heavy chain) and Y91,G92, W96, Y32 (light chain) (Figure 1C,F). Other significant interactions are formed by Y489 of the RBD with the heavy chain sites C97, V52, A33 as well as by F456 of the RBD and the heavy chain positions T30, S31 S32,V52, G53, S54, (Figure 1C,F). Structural analysis of the antibody–RBD interfaces also highlighted contacts formed by Y473, A475, and N487 in the central interface area while other important sites, T478 and Q493, which are among the Omicron-targeted positions, are located on the periphery of the biding interface.

**Figure 2:**
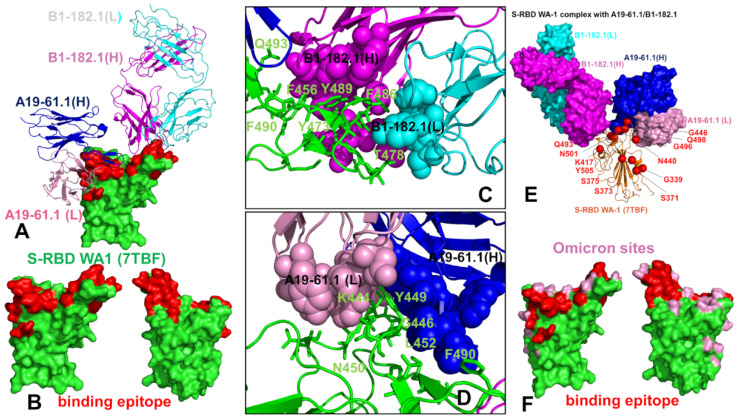
Structural organization of the SARS-CoV-2 RBD complex with the combination of A19-61.1/B1-182.1 antibodies. (**A**) The cryo-EM structure of the S-RBD WA1 complex with the A19-61.1/B1-182.1 pair. The S-RBD is in green, the binding epitope residues are colored in red. The heavy chain of B1-182.1 is in magenta and the light chain in cyan. The heavy chain of A19-61.1 is in blue and the light chain is in pink. (**B**) The S-RBD is shown in two orientations in green with the binding epitope residues in red. (**C**,**D**) A closeup of the interacting residues in the binding interface of the S-RBD complex with B1-182.1 and A19-61.1, respectively. The S-RBD binding residues are shown in green sticks and annotated. The contact sites of the interacting antibodies are shown in spheres colored according to the established color scheme for the heavy and light chain. (**E**) The structure of the S-RBD WA1 complex with A19-61.1/B1-182.1 with the sites of the Omicron mutations. The S-RBD is in orange ribbons, and the Omicron sites are in red spheres and annotated. B1-182.1 is shown in surface colors (heavy chain in magenta, light chain in cyan) and A19-61.1 is also in surface colors (heavy chain in blue, light chain in pink). (**F**) The S-RBD (green) with the binding epitope residues is highlighted in red and sites of the Omicron mutations are in pink.

The structure of the S-RBD WA1 complex with A19-61.1/B1-182.1 antibodies retained a similar binding mode for B1-182.1 and provided an extended binding epitope consisting of the RBD residues K417, L455, F456, K458, Y473, A475, G476, S477, T478, G485, F486, N487, C488, Y489, and Q493 (Figure 2A). The RBD residues involved in the contacts with A19-61.1 included a continuous stretch of residues 440–452 as well as the F490, Q493, S494, and G496 sites. A number of Omicron sites (N440, G446, K417, S477, T478, and Q493) belong to the extended binding epitope (Figure 2A,B). Structural mapping of the binding epitope illustrated the non-overlapping and complementary nature of the binding residues for A19-61.1 and B1-182.1, providing significant coverage of the RBD regions and enabling an effective interference with the RBD–ACE2 binding. A detailed inspection of the intermolecular interactions reinforced the conserved nature of the contacts between the RBD residues F456, F486, and Y489 and hydrophobic sites in the heavy and light chains of B1-182.1 (Figure 2C). The key RBD contacts with B1-182.1 are preserved in the S-RBD WA1 complex with A19-61.1/B1-182.1 and are similarly mediated by F486 (Figure 2C).

The peripheral interactions formed by the T478 and Q493 residues are also maintained in the complex, which reflects a considerable structural similarity to the S-RBD WA1 complexes with a single B1-182.1 and A19-61.1/B1-182.1 combination. The binding interface of A19-61.1 is determined by K444, G446, and Y449 residues that penetrate into a cavity formed by the heavy chain residues H109, N112, I102, and V104 and the light chain residues S31, W32, and D50 (Figure 2D). Noticeably, the functionally important Q493 residue interacts with both B1-182.1 and A19-61.1 antibodies, suggesting the potential importance of this position in the stability of the complex and allostery. Indeed, Q493 forms interactions with G53, G55, and N56 of the B1-182.1 heavy chain, and with A105 and T107 of the A19-61.1 heavy chain (Figure 2C,D). Other important contacts with A19-61.1 are mediated by N450, L452, and F490 (Figure 2D). Structural maps of the binding epitope residues and sites of Omicron mutations in the complex (Figure 2E,F) showed that the Omicron positions are generally either on the edges of the binding epitope or outside of the epitope regions, with the exception of the G446 position that at the core of the binding interface.

**Figure 3:**
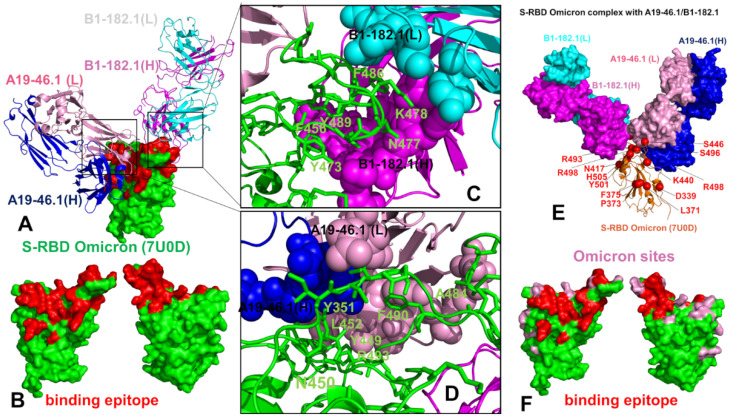
Structural organization of the SARS-CoV-2 RBD Omicron complex with the combination of A19-4.1/B1-182.1 antibodies. (**A**) The cryo-EM structure of the S-RBD WA1 complex with A19-46.1/B1-182.1 pair. The S-RBD is in green and the binding epitope residues are colored in red. The heavy chain of B1-182.1 is in magenta and the light chain in cyan. The heavy chain of A19-46.1 is in blue and the light chain is in pink. (**B**) The S-RBD is shown in two orientations in green with the binding epitope residues in red. (**C**,**D**) A closeup of the interacting residues in the binding interface of the S-RBD complex with B1-182.1 and A19-46.1, respectively. The S-RBD binding residues are shown in green sticks and annotated. The contact sites of the interacting antibodies are shown in spheres colored according to the established color scheme for the heavy and light chain. (**E**) The structure of the S-RBD WA1 complex with A19-46.1/B1-182.1 with the sites of the Omicron mutations. The S-RBD is in orange ribbons, and the Omicron sites are in red spheres and annotated. B1-182.1 is shown in surface colors (heavy chain in magenta, light chain in cyan) and A19-61.1 is also in surface colors (heavy chain in blue, light chain in pink). (**F**) The S-RBD (green) with the binding epitope residues highlighted in red and the sites of the Omicron mutations in pink.

The cryo-EM structure of the S-RBD Omicron with the A19-46.1/B1-182.1 combination revealed a similar binding mode for B1-182.1 as in the S-RBD/B1-182.1 complex, while highlighting a more specific orientation and angle of approach for the A19-46.1 antibody (Figure 3A,B). The binding epitope residues for B1-182.1 in the S-RBD Omicron complex with A19-46.1/B1-182.1 (Figure 3A,B) are essentially identical to those in the S-RBD WA1 complex with A19-61.1/B1-182.1 (Figure 2A,B). It can be noticed that the binding epitope for A19-46.1 has a substantial overlap with A19-61.1 but also featured a stretch of RBD residues 345-354, as well as residues D420, Y421, I468, S469, I470, E471, and A484 in the binding epitope that are specific for A19-46.1 (Figure 3A,B). The primary contacts formed by S-RBD Omicron with B1-182.1 are similarly mediated by the hydrophobic sites F456, F486, and Y489, particularly between F486 and heavy chain positions A33, P95, and D100 and light chain residues S31, Y32, Y91, and W96 (Figure 3C). The interaction contacts also engage Y473, N477, and K478 residues in binding with B1-182.1 (Figure 3C). The dominant contacts with the A19-46.1 antibody are mediated by the Y449 and F490 residues interacting with multiple sites in both the heavy and light antibody chains (Figure 3D).

Additional interaction contacts feature RBD residues Y351, N450, L452, and Q493. The important new contacts formed by the S-RBD Omicron with A19-46.1 are established by the functionally important Omicron position A484 with the light chain residues Y33 and S26 of A19-26.2 (Figure 3D). Interestingly, a number of Omicron sites belong to or are located on the edges of the extended binding epitope, including K440, S446, K417, N477, K478, A484, R493, and S496 (Figure 3E,F). Overall, structural analysis of the S-RBD WA1 and Omicron complexes with a panel of antibodies revealed a complex pattern of binding interactions and binding interfaces that are often dominated by nonpolar interactions formed by a group of hydrophobic residues—Y449, Y453, L455, A475, F456, F486, Y489, and F490—that are important for RBD stability and binding with the host receptor, and where mutations occur only at a very low frequency (<0.05%) [56].

The central question addressed in our study is how these ultra-potent antibodies manage to mitigate mutational escape by the Omicron variant and elicit their neutralization potential given the noticeable presence of the Omicron positions in the binding epitope. We performed a comprehensive computational analysis and mutational profiling of the Omicron RBD–antibody complexes to characterize the dynamic, energetic, and allosteric factors that can be associated with the antibody escape mechanism and explain the experimentally observed mutational escape patterns.

### 3.2. MD Simulations and Distance Fluctuation Analysis of Conformational Ensembles of the S-RBD Complexes with Antibodies Reveal Specific Dynamic Signatures and Stability Centers

**Figure 4:**
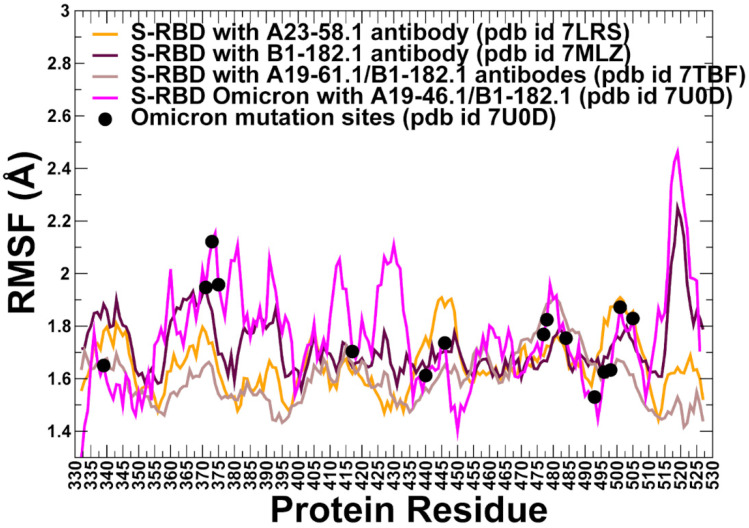
Conformational dynamics profiles obtained from simulations of the SARS-CoV-2 S-RBD complexes. The RMSF profiles for the RBD residues obtained from MD simulations of the S-RBD WA1 complex with A23-58.1 (orange lines), S-RBD with B1-182.1 (maroon-colored lines), S-RBD with A19-16.1/B1-182.1 (light brown lines), and S-RBD Omicron with A19-46.1/B1-182.1 (magenta colors). The positions of the Omicron sites are mapped on the dynamics profile of the S-RBD Omicron with A19-46.1/B1-182.1 and shown as black-filled circles.

All-atom MD simulations were performed for the S-RBD WA1 and S-RBD Omicron complexes with the antibodies. Conformational dynamics profiles are described using the root mean square fluctuations (RMSF) obtained from simulations (Figure 4). The conformational mobility distribution for the S-RBD complex with A23-58.1 and B1-182.1 were similar, showing several deep local minima corresponding to residues 374–377, the RBD core residue cluster (residues 396–403), residues 445–456 that contain β-sheet β5 (residues 451–454) (Figure 4). We observed that the mobile flexible RBM loops (residues 473–487) become partly constrained in the complex, owing to the stabilizing contacts with B1-182.1 antibody that reduce local fluctuations in this region. Noticeably, residues 470–480 retain a certain degree of flexibility in the complex despite forming a portion of the intermolecular interface. At the same time, residues 481–495 experience only minor fluctuations. The RBM residues 501–510 that are exposed to solvent showed more significant displacements. A similar pattern of thermal fluctuations was observed in simulations of the S-RBD complex with A19-61.1/B1-182.1 pair (Figure 4). The most stable RBD positions corresponded to the RBD core residue clusters (residues 396–403 and 430–435). Interestingly, the RBD residues 440–452 involved in the contacts with A19-61.1 showed minor fluctuations, while the binding epitope region 470–485 retained a moderate mobility, and the RBD binding interface sites 486–493 showed reduced fluctuations (Figure 4). The most interesting and intriguing pattern of the RBD dynamics was observed in the S-RBD Omicron complex with A19-46.1/B1-182.1 antibodies. The dynamic profile showed a greater mobility of the S-RBD regions outside of the binding interface (residues 360–395). At the same time, the dynamic profile featured a number of deep local minima in the specific regions, highlighting stabilization of residue clusters 395–403, 420–423, 448–452, and 490–493 (Figure 4). By mapping positions of the Omicron mutations on the dynamics profiles, it could be seen that S371L, S373P and S375F positions experience more appreciable fluctuations, while the reduced mobility in K417N, N440 and G446S may be attributed to the stabilizing interactions with A19-46.1/B1-182.1 antibodies. Despite the intermolecular contacts, the RBM tip positions S477N, T478K remained moderately flexible, but the Omicron positions Q493R and G496S are involved in strong interactions with the antibodies and correspond to well-defined local minima of the dynamics profile signaling their considerable stabilization in the complex (Figure 4). The important implication of these observations is that conformational plasticity of the S-RBD Omicron can be retained in the complex with the antibodies, yielding a rather narrow range of highly stabilized sites induced by binding which could potentially limit the repertoire of resistant mutations.

By monitoring evolution of the interfacial residue-residue contacts from MD simulations for each of the studied RBD–antibody complexes, we evaluated the chemical character and composition of the stable contacts forming at the interface, attempting also to relate this analysis with the experimentally dissociation constants (Table 2). A23-58.1 and B1-182.1 single antibodies target the supersite with only minimal overlap with mutational hotspots (K417, L452R, E484, S494P, and N501). Binding of these antibodies are primarily driven by hydrophobic contacts with the aromatic residues F456, F486, and Y489, which provide nearly 50% of the binding epitope. For both antibodies the average number of stable interfacial contacts mediated by the charged residues is moderate and involve contacts with K417, K458, K478, and Q493 residues of the S-RBD (Table 2). The key residues K417 and E484 are located on the edge of the binding epitope and contribute only ~5% of the binding surface. At the same time, the contribution of polar contacts is more significant. Despite a remarkably similar binding mode for these two antibodies, we found a consistently increased number of stable interfacial contacts for the S-RBD complex with the B1-182.1 antibody. This partly reflects the larger binding epitope for B1-182.1 (~820 Å^2^) as compared to A23-58.1 (~625 Å^2^). The analysis showed the increased number of stable contacts with B1-182.1 that are mediated by charged residues K417 and K458 and the greater contribution of the electrostatic interactions. By employing a simple contact-based predictor of the binding affinity Prodigy [108], we also evaluated the binding free energy for these S-RBD complexes, revealing the enhanced binding affinity of B1-182.1 (ΔG_bind_ = −10.3 kcal/mol) as compared to ΔG_bind_ = −8.5 kcal/mol for A23-58.1 (Table 2). A good agreement between the computed and the experimental affinity determined in the original binding kinetics studies [51] was observed in this comparison. The analysis of the interfacial contacts in the S-RBD and S-RBD Omicron complexes with the synergistic antibody pairs showed an appreciable rise in the number of stable interactions formed by the charged residues (Table 2). Of particular importance are the multiple stable contacts mediated by the S-RBD Omicron residues K444, K458, K478, R346, and R493. Our analysis supports the notion that the electrostatic interactions could play an important stabilizing role in the S-RBD complexes with ultrapotent antibodies [109,110]. Recent quantitative assessment of the antibody escape showed that the S-RBD Omicron variant can be resistant to most neutralizing antibodies, primarily due to either unfavorable or only partly optimized electrostatic interactions [111,112]. Indeed, according to the experiments [51,52], the escaping mutations for the studied ultrapotent antibodies can emerge in the charged positions K444 and R493 of the S-RBD Omicron, causing a modest ~5–7-fold decrease in neutralization, attributed to only partially optimized electrostatic contacts. Consistent with the related studies [109,110,111,112], our analysis similarly indicated that potent antibodies against S-RBD Omicron should be enriched with negatively charged residues in positions interacting with the Omicron positively charged mutational sites. At the same time, a far more significant increase was seen in the number of hydrophobic contacts in the S-RBD and S-RBD Omicron complexes with the synergistic antibody pairs (Table 2). This analysis suggested that the hydrophobic interactions with the conserved RBD sites combined with the favorable electrostatic contributions may be dominant factors driving the improved binding affinities for the examined ultrapotent antibodies capable of neutralizing the Omicron spike variant. In general, the ensemble-based analysis of the interfacial residue-residue contacts revealed the chemical nature of the most relevant binding interactions, also highlighting the connection between the major interaction patterns and potential antibody escape mechanisms.

**Figure 5:**
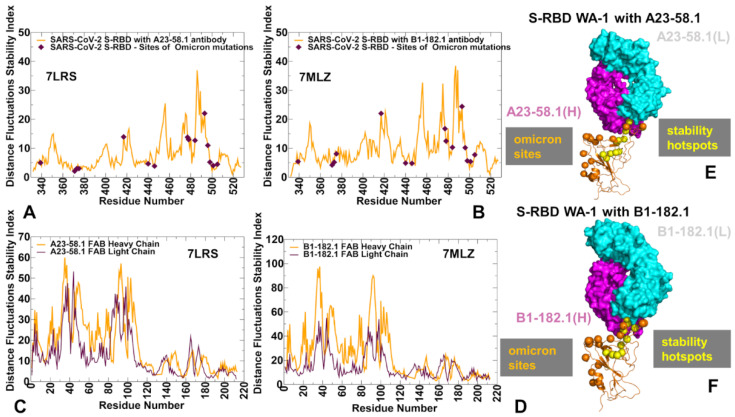
The distance fluctuation stability index profile obtained from simulations of the SARS-CoV-2 S-RBD complexes with A23-58.1 and B1-182.1 antibodies. (**A**) The stability index profile (shown in orange lines) for the RBD residues obtained from MD simulations of the S-RBD WA1 complex with A23-58.1 (pdb id 7LRS). The index values for the sites of the Omicron mutations are highlighted by maroon-colored, filled diamonds. (**B**) The distance fluctuation stability profile for the A23-58.1 antibody (heavy chain in orange lines and light chain in maroon lines). (**C**) The stability index profile (shown in orange lines) for the RBD residues obtained from MD simulations of the S-RBD WA1 complex with B1-182.1 (pdb id MLZ). The index values for the sites of these Omicron mutations are in maroon-colored, filled diamonds. (**D**) The stability profile for the B1-182.1 antibody (heavy chain in orange lines and light chain in maroon lines). (**E**,**F**) Structural maps of the stability centers for the S-RBD complexes with A23-58.1 and B1-182.1 antibodies, respectively. The S-RBD is in orange ribbons. The heavy chains of the antibodies are shown in magenta and the light chains cyan colored. The stability hotspots are shown in yellow-colored spheres. For comparison, the sites of the Omicron mutations are highlighted in orange spheres.

**Figure 6:**
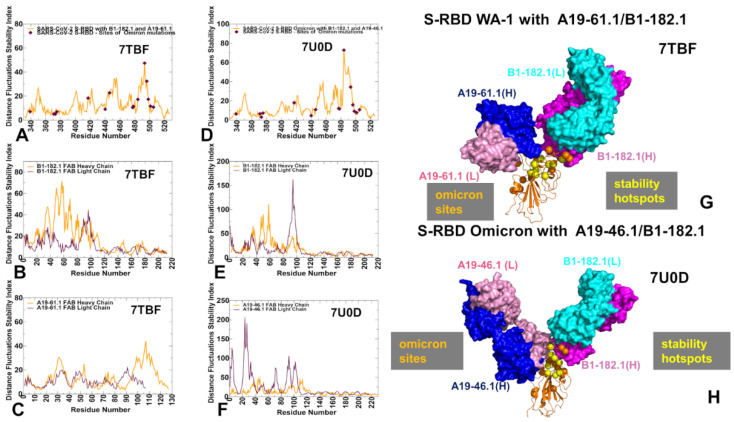
The distance fluctuation stability index profile obtained from simulations of the SARS-CoV-2 S-RBD complex with A19-61.1/B1-182.1 and the S-RBD Omicron complex with A19-46.1/B1-182.1 (**A**) The stability index profile (shown in orange lines) for the RBD residues obtained from the MD simulations of the S-RBD WA1 complex with A19-61.1/B1-182.1 (pdb id 7TBF). The index values for the sites of Omicron mutations are highlighted by maroon-colored, filled diamonds. (**B**) The distance fluctuation stability profile for the B1-182.1 antibody in this complex (heavy chain in orange lines and light chain in maroon lines). (**C**) The distance fluctuation stability profile for the A19-61.1 antibody in this complex (heavy chain in orange lines and light chain in maroon lines). (**D**) The stability index profile (shown in orange lines) for the RBD residues obtained from MD simulations of the S-RBD Omicron complex with A19-46.1/B1-182.1 (pdb id 7U0D). The index values for the sites of the Omicron mutations are in maroon-colored, filled diamonds. (**E**) The stability profile for the B1-182.1 antibody in this complex (heavy chain in orange lines and light chain in maroon lines). (**F**) The stability profile for A19-46.1 in this complex (heavy chain in orange lines and light chain in maroon lines). (**G**) Structural map of the stability centers for the S-RBD WA1 complex with A19-61.1/B1-182.1. (**H**) Structural map of the stability centers for the S-RBD Omicron complex with A19-46.1/B1-182.1. The S-RBD is in orange ribbons. The heavy chain of A19-61.1 and A19-46.1 is in blue and the light chain is in pink. The stability hotspots are shown in yellow-colored spheres and the sites of the Omicron mutations are highlighted in orange spheres.

Using conformational ensembles of the S-RBD complexes, we then computed the fluctuations of the mean distance between each residue and all other protein residues. The resulting distance fluctuation stability indexes measure the energetics of the residue deformations and can point to the regions of protein stability and flexibility (Figure 5 and Figure 6). The high values of the distance fluctuation indexes are typically associated with globally stable residues as they display small fluctuations in their distances to all other residues in the protein system, while small values of this parameter would correspond to the flexible sites that experience large deviations in their inter-residue distances. We first analyzed the distributions of the S-RBD WA1 complexes with A23-58.1 (Figure 5A,B) and B1-182.1 (Figure 5C,D). We observed several dominant and common peaks reflecting similarity to the topological and dynamical features of these S-RBD complexes. The RBD profiles for both complexes revealed a consistent pattern of local maxima that are aligned with the antibody-interacting positions K417, Y421, Y453, L455, F456, Y473, F486, N487, and Y489 (Figure 5A,C). Strikingly, the most dominant stability hotspots in the S-RBD complexes with A23-58.1 and B1-182.1 coreponded to the conserved hydrophobic positions L452, L455, F456, Y473, F486, Y489, F490, and L492 (Figure 5A,C). The stability peaks for the interacting antibodies A23-58.1 (Figure 5B) and B1-182.1 (Figure 5D) corresponded to the heavy chain positions T30, S31, and S32 interacting with F456 on the RBD, and the heavy chain residues 90–95 forming contacts with the F486 position of the RBD. Our findings agree with the structural analysis of the most potent antibodies against Omicron sublineages BA.1 and BA.2, showing that their high binding affinity and neutralizing activity are driven by targeting this very group of conserved and RBD stability-essential sites [113,114,115].

Structural map of the distribution peaks along with the Omicron positions (Figure 5E,F) illustrated the preferential localization of the stability centers in the RBD core and the immobilized region of the binding interface, while Omicron sites are located in the more flexible RBD regions. Together, the structural and dynamics analyses indicated that the L455, F456, Y473, F486, N487, and Y489 residues become further rigidified in the complexes, largely due to their vital role in the binding interface (Figure 5A,B). These results are consistent with the functional experiments in which the cell surface-expressed spike binding to A23-58.1 and B1-182.1 was knocked out by F486R, N487R, and Y489R mutations, completely abolishing neutralization [51]. Furthermore, for A23-58.1, there are several escape mutants, including F486 mutations, and particularly F486S, while B1-182.1 escape can be mediated by the F486L mutant [51]. Importantly, residues F486, N487, and Y489 were present in >99.96% of sequences, and only F486L was noted in the database at >0.01% (0.03%) [51]. Hence, the conformational dynamics and distance fluctuation analysis revealed that the L452, L455, F456, Y473, F486, Y489, F490, and L492 hydrophobic sites correspond to the protein stability hotspots, suggesting that the repertoire of antibody escape mutations in these sites may be extremely limited as it would incur a significant stability fitness cost. These findings are consistent with the recent studies showing that residues Y449, L452, L455, E484, Y489, F490, L492, Q493, and S494 can be among the immune-escaping hotspots that may destabilize binding with antibodies and erode neutralizing immune responses [116]. Furthermore, mutations of the common hydrophobic hotspots (Y449, Y473, L455, F456, and Y489) can disrupt both the stability of the RBD and binding to ACE2 and ultra-potent neutralizing antibodies [115]. Hence, mutations of these hotspots can compromise the balance of multiple fitness trade-offs of the virus, specifically between immune escape, RBD stability, and the affinity with the ACE receptor. Another important revelation of the distance fluctuation analysis is that the Omicron sites displayed only moderate or low stability indexes, which reflected conformational flexibility of these residues even when they engage in the interactions with the antibodies (Figure 5A,B). It is also worth pointing out that only a single Omicron position, Q493, displayed a moderate-high value in the stability index, indicating that substitutions in this site may potentially produce antibody-escaping mutants. Consistent with the predicted moderate stability effect, only marginal B1-182.1 escape can be mediated by Q493R but with minor impact on binding and no appreciable reduction of the neutralization activity [51].

The distance fluctuation stability profile for the S-RBD complex with the A19-61.1/B1-182.1 antibody combination showed an overall similar profile, reinforcing the notion that a subset of stability hotspots on the RBD may be preserved across the S-RBD complexes (Figure 6A). The major stability profile peaks corresponded to the hydrophobic sites V350, V401, I402, L455, F456, Y473, A475, F486, and Y489 (Figure 6A). In addition, functional positions Q493 and S494 corresponded to the largest peak in the distance fluctuation profile, reflecting antibody-induced stabilization of these sites that are more flexible in the unbound form. Moreover, the Q493 residue interacts with both B1-182.1 and A19-61.1 antibodies, and the increased stabilization of this site may be also associated with its potential mediating role in the long-range intermolecular interactions in the complex. Interestingly, several A19-61.1-interacting positions, such as G446 and L452, featured among the moderate local peaks of the distribution (Figure 6A). In this context, it is worth noting that A19-61.1 escape mutations include G446V/S and S494R that occur at the exceptionally low frequency [51,52], which is consistent with the role of these sites as the protein stability hotspots in the complexes. The distance fluctuation profile for the B1-182.1 antibody showed strong peaks for residues 50–52 of the heavy chain (Figure 6B) that interacts with the RBD stability centers F486, Y489, and F490. The distribution for the A19-61.1 antibody displayed the largest peak for residues 107–112 of the heavy chain (Figure 6C) that interface with the K444, G446, Y449, and L452 positions on the RBD. The observed distribution patterns for the interacting antibodies A19-61.1 and B1-182.1 reflected stabilization of the key intermolecular interfaces and have a mediating role in the RBD stability centers G446, Y449, L452, F486, Y489, and F490. These positions are often targeted by class I and II antibodies. Indeed, the most frequently targeted RBD residues by class I antibodies include L455, F456, F486, N487, and Y489, while for class II antibodies the most targeted sites are Y449, G485, and F486 [117].

In the S-RBD Omicron complex with A19-46.1/B1-182.1 antibodies, there was some redistribution of the profile peaks detected in the N450, Y451, L452, L455, A484, and F490 positions (Figure 6D). Most of the distribution peaks are associated with the binding interface residues of A19-46.1 while the stability index values for the B1-182.1-interacting positions (Y473, L455, R493, N477, K478, G485, F486, N487, and Y489) are more moderate (Figure 6D). Of interest is that the Omicron mutational sites, with the exception of E484A and Q493R positions, displayed moderate stability indexes (Figure 6D). While E484A mutation can decrease the stability of the unbound S-RBD, binding with the A19-46.1 antibody in the S-RBD Omicron complex may favor this position as a mediating stability center (Figure 6D). This is in line with the functional data showing that the E484A mutation did not affect the neutralization capacity of A19-46.1 [52]. The limited repertoire of escape mutations for A19-46.1 is associated with the predicted stability hotspot sites N450, L452, and F490 (Figure 6D). Notably, among the mutations mediating escape to A19-46.1, only F490L and L452R are present in the GISAID sequence database at a greater than 0.01% frequency [56]. The distance fluctuation profile of the B1-182.1 antibody in the S-RBD Omicron complex displayed the highest peaks for the light chain residues L92, Y93, and M94 that interface with the A484 and F486 RBD sites (Figure 6E), while the A19-46.1 antibody showed strong peaks for residues L24, S25, and S26 of the light chain of the antibody that pack directly against A484 on the RBD (Figure 6F). In addition, some less significant stability peaks were seen for the heavy chain residues L105, L106, P107, of A19-46.1 (Figure 6F) packed against the RBD hotspots N450, Y451, and L452. Hence, in the S-RBD Omicron complex with A19-46.1/B1-182.1, one hotspot cluster is centered around the A484 position interfacing with both antibodies, while several additional stability centers are mediated by the N450, L452, and F490 positions that are engaged in binding with the A19-46.1 antibody. Another important finding of our analysis is that the Omicron mutational sites in these complexes typically featured small distance fluctuation indexes indicative of the conformational adaptability of these residues. Structural mapping of the stability centers and Omicron mutation sites (Figure 6G,H) highlighted only small overlap between these groups. In particular, for the S-RBD Omicron complex with A19-46.1/B1-182.1, the structural projection of the stability centers mimicked a “pathway” that connects the RBD core with the RBM residues serving as “bridges” between the synergistically acting antibodies. It may be argued that this structural disposition of the stability centers is associated with their role in mediating the long-range communications in the complex. In some contrast, Omicron mutations are broadly distributed on the RBD binding interface and create a dynamic “shield” surrounding the protein stability regions.

Combined, the results of the conformational dynamics and distance fluctuation analysis showed that the protein stability centers in the S-RBD complexes often corresponded to the antibody-specific, immune-escaping hotspots. Importantly, the common stability hotspots—Y449, Y473, L455, F456, F486, and Y489—in these complexes are constrained by the requirements for the RBD folding and binding with the ACE2 host receptor, and therefore may be limited in evolving antibody-escaping variants. Hence, protein stability signatures mediated by the antibody binding can affect the pattern of mutational escape and be one of the important contributing factors that determines mechanisms of immune evasion.

### 3.3. Ensemble-Based Mutational Scanning and Energetic Cartography Identifies Binding Affinity Hotspots in the SARS-CoV-2 RBD Complexes with Ultrapotent Antibodies

**Figure 7:**
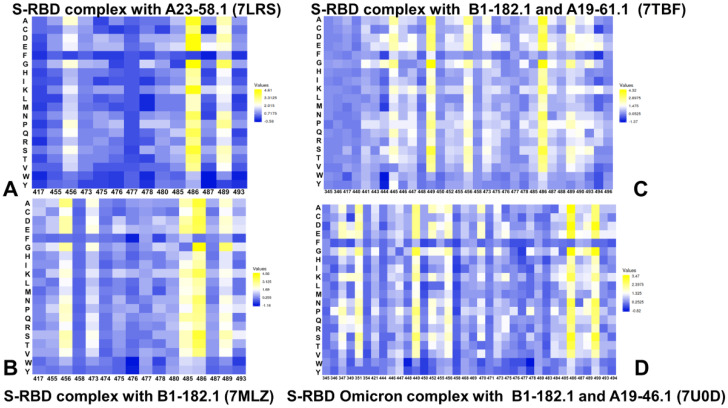
The ensemble-based mutational scanning of binding for the SARS-CoV-2 S-RBD and S-RBD Omicron complexes with ACE2. The mutational scanning heatmap for the binding epitope residues in the S-RBD WA1 complex with A23-58.1 (**A**), the S-RBD WA1 complex with B1-182.1 (**B**), the S-RBD WA1 complex with the A19-61.1/B1-182.1 combination (**C**), and the S-RBD Omicron complex with A19-46.1/B1-182.1 (**D**). The binding energy hotspots correspond to residues with high mutational sensitivity. The heatmaps show the computed binding free energy changes for 20 single mutations on the sites of variants. The squares on the heatmap are colored using a 3-colored scale—blue/white/yellow—with yellow indicating the largest unfavorable effect on stability. The standard errors of the mean for binding free energy changes were based on a different number of selected samples from a given trajectory (500 and 1000 samples), and are within 0.07–0.16 kcal/mol.

By employing the conformational ensembles of the S-RBD WA1 and S-RBD complexes with antibodies, we performed comprehensive mutational scanning of the interfacial RBD residues and computed binding free energy changes. In silico mutational scanning was done using the BeAtMuSiC approach [97,98,99]. This approach allows for accurate predictions of the effect of mutations on the binding affinity and the protein stability of the complex. To provide a comparison between the computational and experimental data, we constructed mutational heatmaps for the RBD binding interface residues (Figure 7). Intriguingly, despite structural similarities and common binding epitopes, binding heatmaps for the S-RBD WA1 complex with A23-58.1 (Figure 7A) and S-RBD Omicron complex with B1-182.1 (Figure 7B) displayed appreciable differences and featured several unique mutational signatures. In the S-RBD WA1 complex with A23-58.1, a fairly elevated level of mutational tolerance to the binding interactions was observed for many epitope residues, including K417, L455, Y473, A475, S477, T478, G485, and Q493 residues (Figure 7A). At the same time, the mutational heatmap clearly identified three major binding hotspots in the F456, F486, and Y489 positions, where particularly large destabilization changes were induced by mutations of the F486 residue (Figure 7A). These results agree with the experimental data, showing that the binding and neutralization capacity of A23-58.1 can be markedly reduced by mutations in F486 and Y489 (particularly F486R/S, Y489R) and partly impaired by some mutations in F456 (F456R/Q/S) [51]. Consistent with the experiments, our energetic analysis showed that the F486Q, F486R, and F486S mutations yielded the most significant destabilization: ΔΔG = 3.74 kcal/mol, ΔΔG = 3.75 kcal/mol, and ΔΔG = 4.04 kcal/mol, respectively (Figure 7A). According to the mutational scanning analysis, mutations in the Omicron sites S477, T478, and Q493 caused only moderate destabilization changes, with S477N, T478K, and Q493R substitutions yielding ΔΔG = 0.14 kcal/mol, ΔΔG = 0.73 kcal/mol, and ΔΔG =0.33 kcal/mol, respectively, for the A23-58.1 antibody (Figure 7A). This is consistent with the observed tolerance of the antibody binding to mutations in these Omicron positions [51].

**Figure 8:**
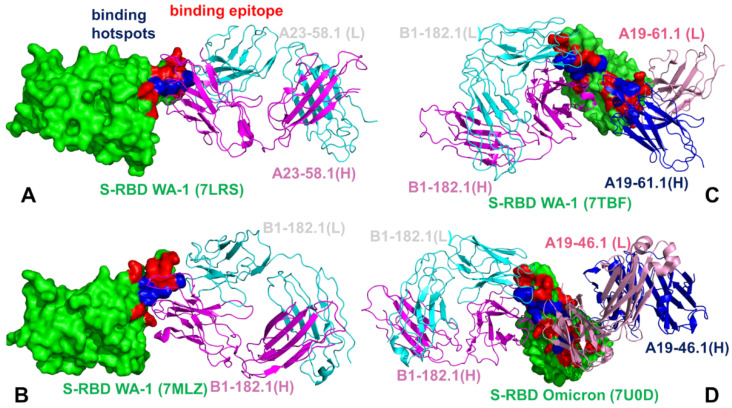
Structural maps of the binding epitope residues and binding energy hotspots in the S-RBD WA1 complex with A23-58.1 (**A**), the S-RBD WA1 complex with B1-182.1 (**B**), the S-RBD WA1 complex with the A19-61.1/B1-182.1 combination (**C**), and the S-RBD Omicron complex with A19-46.1/B1-182.1 (**D**). The S-RBD is in green, the binding epitope residues are colored in red, and the binding hotspots are colored in blue. The heavy chain of A23-58.1 and B1-182.1 on panels (**A**–**D**) is in magenta and the light chain in cyan. The heavy and light chains of A19-61.1 (panel **C**) and A19-46.1 (panel **D**) are in blue and pink ribbons, respectively.

The mutational heatmap of the S-RBD Omicron complex with B1-182.1 (Figure 7B) displayed a more sensitive profile of energetic changes, pointing to binding hotspots in the F456, Y473, G485, F486, Y489, and Q493 positions. Common to both complexes, the most sensitive RBD positions corresponded to the F456, F486, and Y489 sites (Figure 7A,B). However, we observed a greater binding sensitivity to mutations in the F456 and Q493 sites. Our data revealed that the F486Q, F486R, and F486S mutations yielded the largest destabilization changes (ΔΔG = 2.95 kcal/mol, ΔΔG = 2.55 kcal/mol, and ΔΔG = 3.92 kcal/mol, respectively) that were moderately less detrimental as compared to their effect on binding with A23-58.1. Mutations of S477, T478, and Q493 similarly produced small energetic changes where Omicron mutations S477N, T478K, and Q493R yielded ΔΔG = 0.26 kcal/mol, ΔΔG = 1.02 kcal/mol, and ΔΔG = 0.60 kcal/mol, respectively (Figure 7B). Structural maps of the binding epitope residues and hotspots for these S-RBD complexes (Figure 8A,B) illustrated a remarkable similarity in the binding modes and binding epitope residues. The structural mapping also highlighted differences between the binding energy hotspots and sites of the Omicron mutations. It is evident that both A23-58.1 (Figure 8A) and B1.182.1 antibodies (Figure 8B) target a focused RBD tip region with only a small overlap with the Omicron positions.

A more complex picture emerged from the mutational heatmaps of the S-RBD complex with B1-182.1/A19-61.1 (Figure 7C) and the S-RBD Omicron complex with B1-182.1/A19-46.1 (Figure 7D). In both complexes, the key binding hotspots corresponded to the Y449, F456, F486, Y489, and F490 positions. Of particular significance is the fact that the conserved hydrophobic residues Y449, Y453, L455, F486, Y489, and F490 consistently emerged not only as the key stability centers but also as the dominant binding hotspots in the studied complexes (Figure 7). Structural maps of the binding interfaces for these complexes (Figure 8C,D) highlighted the extended binding epitope induced by the antibody pairs, also illustrating the binding hotspot clusters interacting with each of these antibodies. These findings are consistent with the recent studies showing that these residues can destabilize binding with antibodies but at the unacceptable functional cost of disrupting the balance between various fitness tradeoffs, perturbing the intrinsic RBD stability and compromising binding to ACE2 [116]. These factors may narrow the “ evolutionary path” for the virus to adopt escape mutations in these key binding hotspots, thereby allowing for the cross-reactive antibodies to retain their neutralization efficiency. We also observed a strong binding sensitivity of A19-61.1 to mutations in the G446 and Q493 positions, particularly Omicron mutations G446S and Q493R (Figure 7C). These results are in excellent agreement with the pioneering structure–function experiments [51,52] that emphasized the role of the G446S mutation causing a complete loss in activity for A19-61.1 against the Omicron variant. The energetic heatmap analysis of the S-RBD Omicron complex with A19-46.1/1-182.1 (Figure 7D) detailed the mutational sensitivity of residues Y449, N450, L452, and F490 that are targeted by escape mutations Y449S, N450S, N450Y, and L452R [51,52].

### 3.4. Allosteric Mutational Profiling of the Interaction Networks in the S-RBD Complexes Discern Sites and Mechanisms of Mutational Escape

**Figure 9:**
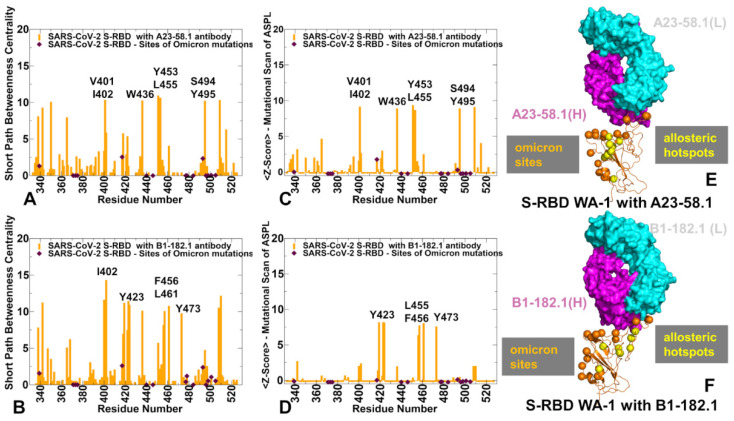
The network-based analysis of the S-RBD complexes with A23-58.1 and B1-182.1 antibodies. (**A**) The SPC centrality profile (shown in orange bars) for the RBD residues in the S-RBD WA1 complex with A23-58.1 (pdb id 7LRS). (**B**) The SPC centrality profile (shown in orange bars) for the RBD residues in the S-RBD WA1 complex with B1-182.1 (pdb id 7MLZ). The SPC values for the sites of Omicron mutations are highlighted by maroon-colored, filled diamonds. (**C**) The Z-score ASPL profile (shown in orange bars) for the RBD residues in the S-RBD WA1 complex with A23-58.1. (**D**) The Z-score ASPL profile for the RBD residues in the S-RBD WA1 complex with B1-182.1. (**E**,**F**) Structural mapping of the allosteric centers associated with the Z-score ASPL peaks for the S-RBD complexes with A23-58.1 and B1-182.1, respectively. S-RBD is in orange ribbons. The heavy chain for the antibodies is in magenta and the light chain is in cyan. The allosteric hotspots are in yellow spheres and sites of the Omicron mutations are in orange spheres.

**Figure 10:**
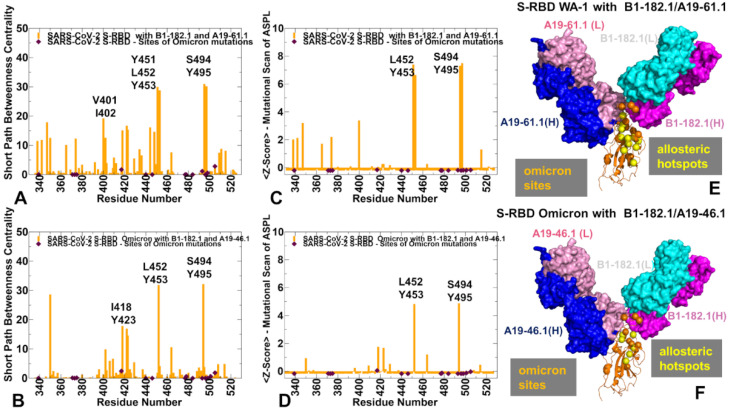
The network-based analysis of the S-RBD WA1 and S-RBD Omicron complexes with antibody combinations. (**A**) The SPC centrality profile (shown in orange bars) for the RBD residues in the S-RBD WA1 complex with A19-61.1/B1-182.1 (pdb id 7TBF). (**B**) The SPC centrality profile (shown in orange bars) for the RBD residues in the S-RBD Omicron complex with A19-46.1/B1-182.1(pdb id 7MLZ). The SPC values for the sites of Omicron mutations are highlighted by maroon-colored, filled diamonds. (**C**) The Z-score ASPL profile (shown in orange bars) for the RBD residues in the S-RBD WA1 complex with A19-61.1/B1-182.1. (**D**) The Z-score ASPL profile for the RBD residues in the S-RBD Omicron complex with A19-46.1/B1-182.1. (**E**,**F**) Structural mapping of the allosteric centers associated with the Z-score ASPL peaks for the respective S-RBD complexes. The allosteric hotspots are shown in yellow spheres and sites of the Omicron mutations are in orange spheres. S-RBD is shown in orange ribbons. The heavy chain for B1-182.1 is in magenta and the light chain is in cyan. The heavy chain for A19-61.1 and A19-46.1 is in blue and the light chain is in pink on panels E and F, respectively.

We performed dynamic network analysis of the conformational ensembles and employed a recently introduced perturbation-based network approach for mutational scanning of allosteric residue potentials [72] to characterize the allosteric interaction networks and identify allosteric hotspots in the S-RBD complexes. In the proposed model, allosteric hotspots are identified as residues in which mutations incur significant edgetic perturbations of the global residue interaction network that disrupt the network connectivity and cause a significant impairment in the global network communications. Using a graph-based network model of the residue interactions in which the network edges between nodes are weighted using dynamic residue–residue correlations obtained from the MD simulations, we computed the ensemble-averaged distributions of several residue-based topological network metrics (Figure 9 and Figure 10). The short path residue centrality (SPC) is used to analyze the modularity and community organization of the dynamic residue interaction networks. The SPC distributions reflect the extent of the residue connectivity in the interaction networks and allow for characterization of the mediating clusters in the complexes. The Z-score betweenness centrality is based on computing the average shortest path length (ASPL) as outlined in detail in the Methods section. By systematically introducing mutational changes in the S-RBD positions and using the equilibrium ensemble of the original system, we reevaluate the dynamic inter-residue couplings and compute mutation-induced changes in the ASPL parameter. These changes are then averaged over all substitutions in a given residue. In this manner, we characterized the average mutational sensitivity of each residue node on the changes in the network modularity and allosteric communications. By identifying residues where mutations on average induce a significant increase in the ASPL metric and therefore have a dramatic effect on the allosteric interaction network, we locate allosteric control points and regulatory hotspots that control long-range communications in the S-RBD complexes.

The SPC distribution for the S-RBD complexes with A23-58.1 (Figure 9A) and B1-182.1 antibodies (Figure 9B) revealed dense clusters of high centralities in the regions that are often aligned with the stability centers. The emergence of local clusters of mediating residues implies a high level of connectivity in the residue interaction network, which may allow for diverse communication routes in the complex. The high centrality peaks in both the S-RBD complex with A23-58.1 corresponded to the hydrophobic sites V350, V401, I402, W436, Y453, L455, S494, and Y495 (Figure 9A). A similar but less dense distribution is seen in the S-RBD complex with B1-182.1 (Figure 9B). The key peaks in this distribution are I402, Y423, F456, L461, and Y473 residues. Interestingly, the mediating clusters of the long-range interactions in these complexes are anchored by a group of residues that only partly overlap with the stability and binding hotspots. We noticed that the Omicron positions K417 and Q493 displayed moderate SPC values, indicating that these sites also can be involved in mediating allosteric interactions. Perturbation-based mutational scanning of allosteric residue propensities provided information about potential allosteric hotspots by mapping a space of network-altering, allosteric, ‘edgetic’ variant sites (Figure 9C,D). In this model, the peaks of the Z-score ASPL profile corresponded to sites where mutational changes can incur a significantly increased ASPL value, which implies a dramatic loss in the network connectivity. Importantly, the major peaks of the SPC and Z-ASPL distributions correspond to the same group of key mediating sites, but the ASPL profile allows to clearly distinguish mutation-sensitive hotspots of allosteric interactions.

Indeed, in the S-RBD complex with A23-58.1, the key allosteric hotspots revealed by the Z-score ASPL profile were aligned with the I402, W436, Y453, L455, S494, and Y495 positions (Figure 9C), while in the complex with the B1-182.1 antibody, the major peaks singled out Y423, L455, F456, L461, and Y473 residues (Figure 9D). In network terms, mutations in these positions could affect multiple intra- and inter-molecular interactions altering the network connectivity, which could adversely affect the long-range allosteric interactions. This analysis revealed several antibody-specific allosteric centers, such as Y453, S494, and Y495, in the complex with A23-58.1, while L461 and Y473 in the complex with the B1-182.1 antibody. Notably, the sites of the Omicron mutations in these S-RBD complexes featured small Z-score ASPL values in both complexes, indicating that allosteric communications between the S-RBD and antibodies may be tolerant to modifications in the Omicron positions (Figure 9C,D). Structural projection of the allosteric centers showed that the RBD positions mediating the network connectivity could form a “pathway” connecting the RBD core with the central part of the binding interface (Figure 9E,F). The Omicron sites occupy more flexible regions and are dynamically coupled with the stable allosteric centers.

In the S-RBD complex with A19-61.1/B1-182.1, the shape of the SPC centrality profile is similar, but the most allosterically sensitive to mutation positions corresponded to the Y451, Y453, S494, and Y495 residues (Figure 10A). These peaks were further accentuated in the Z-score ASPL profile (Figure 10B), indicating that mutations of these residues may perturb network connectivity and alter allosteric interactions in the S-RBD complex.

The results revealed that S494 residue is not only a protein stability center but also an important allosteric mediating hotspot of long-range communications in the complex with A19-46.1/B1-182.1. The experimental data showed that mutations in S494 (S494R and S494P) can mediate a strong escape from A19-61.1 antibody binding [51]. According to our analysis, this may result from the combined effect of multiple factors, including the protein stability reduction, binding affinity loss, and also weakening of the allosteric interactions in the complex. In particular, mutations of S494 can reduce binding by the antibodies while having a minimal effect on the RBD–ACE2 binding [118]. In support of the predicted allosteric role of the S494 position, recent experimental studies showed that the neutralization potential of the potent antibodies may be significantly hampered with the addition of synergetic mutations K417N/N501Y to E484K and E484K/N501Y to S494P [118], suggesting a long-range cooperativity between sites of circulating mutations.

For the S-RBD Omicron complex with A19-46.1/B1-182.1, we found that N450, L452, and S494 are the dominant peaks of the SPC and ASPL distributions (Figure 10C,D) and correspond to potential allosteric hotspots of the interaction network in which mutations can markedly alter the efficiency of the long-range interactions. These positions are also binding epitope residues. While mutational scanning showed that substitutions of these sites induce appreciable binding affinity losses, the binding free energy changes for the experimentally observed escape mutations N450S, N450Y, and L452R [51,52] are not markedly different from mutation-induced destabilization in other epitope sites. Structural mapping of the allosteric centers highlighted the proximity of the stable allosteric centers and Omicron sites, also illustrating the connectivity of the allosteric hotspots linking the RBD core with the intermolecular interfaces (Figure 10E,F).

Overall, this analysis showed that the potent neutralizing antibodies target a privileged group of the RBD residues that form the binding hotspots but also serve as protein stability and allosteric control centers. As a result, the repertoire of antibody escape mutations can be significantly curtailed, as modifications in these positions may compromise the RBD folding stability, affect ACE2 binding, and also alter the long-range interactions and allosteric communication in the S-RBD complexes. Our results suggest that the experimentally observed escape mutants for the studied antibodies may be determined by the cumulative contribution and fitness tradeoffs of the local binding interactions, protein stability requirements, and long-range allosteric effects. These results support the recent biophysical experiments showing that the differences in the mechanical stability of the S-RBD interactions with ACE2 and antibodies may determine fitness advantages and describe more accurately the thermodynamics and kinetics of the binding mechanisms and immune evasion patterns [41,42,43]. Noteworthy, caution needs to be exercised when inferring evidence of a direct causal association between the antibody escape mechanisms and the confluence of the examined molecular factors. The results presented in this study suggest that the antibody escape patterns may be linked with a complex balance of binding, protein stability, and allostery factors, but certainly only indicate the observed association that requires further investigation. The integration with the biophysical and cell-based experiments may help to unveil the important signals of mutational escape at the molecular and cellular level, allowing to quantify the determinants of the immune evasion.

## 4. Conclusions

The integrated computational analysis of the S-RBD Omicron complexes with a set of ultra-potent antibodies revealed several important structural and energetic factors contributing to the mutational escape mechanism. Consistent with the experimental evidence, mutational profiling of binding, protein stability, and allosteric interactions in the S-RBD Omicron complexes revealed that the binding interactions and long-range communications in the complexes are generally tolerant to the Omicron mutations. We found that the neutralizing antibodies against the Omicron variant target the conserved protein stability centers Y449, Y453, L455, F486, Y489, and F490, which emerged as the dominant binding hotspots in the studied complexes. As a result, mutations in these residues that can severely impair binding with the antibodies would also incur an unacceptable functional cost of disrupting the intrinsic RBD stability and compromising binding to ACE2. Perturbation-based mutational scanning of allosteric residue propensities provided information about potential allosteric hotspots by mapping a space of network-altering, allosteric, ‘edgetic’ variant sites. By identifying residues where mutations on average induce a significant increase in the ASPL metric, and therefore have a dramatic effect on the allosteric interaction network, we located allosteric regulatory hotspots that control long-range communications in the S-RBD complexes. We found that some allosteric centers mediating long-range interactions correspond to the important sites of mutational escape—N450, L452, and S494—while only moderately affecting the RBD–antibody binding. Together, the predicted energetic tolerance of the antibody binding to Omicron mutations and the convergence between the binding affinity hotspots, the protein stability centers, and allosteric control points support a strong association between these effects and the observed antibody escape pattens. We suggest that by specifically targeting these universal hotspots, the ultra-potent antibodies could resist the Omicron mutations and limit the “evolutionary path” for the virus to adopt viable escape mutants. In this scenario, the emergence of antibody escape mutations in the binding hotspots may become restricted due to the RBD folding stability constraints, the functional requirements for ACE2 binding, and the regulatory control of the structural RBD transformations. The insights from this investigation suggest therapeutic venues for targeted exploitation of the binding hotspots and allosteric centers that may potentially aid in evading drug resistance.

## Figures and Tables

**Table 1:** Structures of the SARS-CoV-2 S-RBD complexes examined in this study.

PDB	System	1 Simulation	# Simulations
7LRS	RBD/A23-58.1	500 ns	10
7MLZ	RBD/B1-182.1	500 ns	10
7TBF	RBD/A19-61.1/B1-182.1	500 ns	10
7U0D	RBD Omicron/A19-46.1/B1-182.1	500 ns	10

#: The Number of Simulations.

**Table 2:** Ensemble-averaged statistics of interfacial residue–residue contacts in the SARS-CoV-2 S-RBD complexes examined in this study.

Interfacial Contacts	RBD A23-58.1	RBD B1-182.1	RBD A19-61.1/B1-182.1	RBD Omicron A19-46.1/B1-182.1
Charged–charged	3	4	7	8
Charged–polar	7	8	10	13
Charged–apolar	2	5	17	11
Polar–polar	3	4	6	3
Polar–apolar	13	21	27	26
Apolar–apolar	22	24	39	35
ΔG comput. (kcal/mol)	−8.5	−10.3	−12.9	−12.6
Kd (nM) experiment	7.3	2.55	2.33 (A19-61.1)	3.58 (A19-46.1)

## Data Availability

The data presented in this study are fully described in the manuscript.

## Supplementary Materials

The following supporting information can be downloaded at: https://www.mdpi.com/article/10.3390/biom12070964/s1, The python scripts used for calculation of the distance fluctuations.
