# Supplementary material for: Integrating Conformational Dynamics and Perturbation-Based Network Modeling for Mutational Profiling of Binding and Allostery in the SARS-CoV-2 Spike Variant Complexes with Antibodies: Balancing Local and Global Determinants of Mutational Escape Mechanisms
Source: Biomolecules. 2022 Jul 10;12(7):964. doi: 10.3390/biom12070964 (PMC9313167; doi:10.3390/biom12070964)
Supplement: Supplementary file 1 [file biomolecules-12-00964-s001.zip › biomolecules-1799882-supplementary/SUPPLEMENTARY MATERIALS/SUPPLEMENTARY_MATERIALS.pdf]

# Supplementary

# Materials

## Integrating Conformational Dynamics and Perturbation-Based Network Modeling for Mutational Profiling of Binding and Allostery in the SARS-CoV-2 Spike Variant Complexes with Antibodies: Balancing Local and Global Determinants of Mutational Escape Mechanisms

**Gennady Verkhivker,[1,2]* Steve Agajanian[1], Ryan Kassab[1], Keerthi Krishnan[1]**

[1] Keck Center for Science and Engineering, Graduate Program in Computational and Data Sciences, Schmid
College of Science and Technology, Chapman University, Orange, CA 92866, United States of America
[2] Department of Biomedical and Pharmaceutical Sciences, Chapman University School of Pharmacy, Irvine, CA 92618, United States of America

*Correspondence: verkhivk@chapman.edu; Tel.: +1-714-516-4586 (G.V)

Supplemental Materials include scripts for computation of distance fluctuations from MD simulations.
